# Editorial, The Practice of Radiology, Ethical Considerations

**Published:** 2008-11

**Authors:** KP Mody

**Affiliations:** Tata Memorial Hospital, IJR, Vol. VIII. November 1954 No. IV

Soon After the discovery of X-rays by Professor Roentgen in the 1898, medical men and scientist got interested in its potentialities as an aid to the diagnosis of diseases. In those days there was no recognised speciality of Radiology, some of the surgeons and physicians experiment with the crude apparatti then available – coils, gas, tubes, platinum interrupters etc. – used the fluorescent screen and took a few radiograms now and again. These pioneer efforts and the success they met with made medical men at last realise that there was ample of scope for specialisation and radiologists as a class came into existence. It was then recognised that it was not just a side line or a hobby to be indulged in one's spare time but was a full time job requiring all his time and energy. Special radiology departments were organised in many of the hospitals. Even after the speciality had branched out as a distinct entity, for quite a long time the radiologists in charge of the department was merely concerned with the taking of radiograms which were then sent on to the physicians and surgeons who interrupted them in the light of their clinical findings. Except for a few outstanding personalities such as Barclay of England, Haudek of Vienna and a few others in Europe and America, the radiologist functioned as a radiographer, his job was to look after the machines and the technical part of the work.

50 years back medicine was practiced, in what we might call water tight compartments. The surgeons was concerned chiefly with his operations, the physicians, with his stethoscope, was engrossed in writing prescriptions for his sick patients. There was no co-operations as we now understand. Everyone worked in his own solitary sphere and any attempt at discussion or any differences of opinion was looked upon with disapproval. There was no attempt at collaboration, no staff meetings or conferences. Under these circumstances it is easily understandable that interpretation was best left with surgeons and physicians in charge of the case. They had the case in front of them, they were in possession of full notes of the cases so that they could co-relate the x-ray appearances with the clinical findings better than the radiologist who was confined as the solitary figure in the basement with his coils, breaks, gas tubes and such other paraphernalia. He produced pictures and occasionally ventured on a report, which was very often was sketchy and unhelpful, consisting as if did of laborious description of “shadows” “opacities”, “filling defects” etc. Thus it was in the best of the patient for the medical men in charge of their case to evaluate their problems themselves with whatever little assistance that could be given by the radiologist.

This unsatisfactory state of affairs has undergone a radical change since special courses and diplomas were established for the training of radiologists. Cambridge University being the first to introduced the D.M.R.E about the year 1918. The status and the outlook for radiologists have greatly improved since then and far from being considered as technicians they have come in line with the consultants.

Reading and interpretation of radiograms are the prime function of the radiologist but unfortunately the habit of years ingrained and rooted in the minds of our colleagues in other branches of Medicine is rather hard to overcome, some of them still think that it is for them to make the diagnosis.

Interpretation of radiograms entails two distinct functions. Correct evaluation of different shadows and opacities seen in radiograms is essentially the duty of the radiologist, it s for him to decide whether the different appearances seen in the radiograms are within the limits of normality or are signs of disease. Furthermore, the radiologist has to determine to what extent the abnormal appearance visualised on the radiograms fit in with the clinical picture. In making such important decisions the co-operation of our medical colleagues is imperative. In hospital it is easy to secure such collaboration, as a matter of fact in most hospitals in the West, it is the recognised practice for the staff to meet and discuss out the problems of the day with the radiologist. Unfortunately in our country such is not the case, except in few hospitals. Moreover, in private practice, it is obviously difficult to secure the co-operation of the referring doctor. But inspite of these handicaps, it is the prime duty of the radiologist to obtain the clinical and laboratory data before he rushes into a hasty report on the case under a review. A radiogram is like a jig-saw puzzle with radiologist trying to fit in the pieces but without having before him the picture that is represents. It must be emphasized here that the radiologist's report should be precise and to the point and should as far as possible bear the imprint of finality. No effort should be spared to come to a definite diagnosis, if further investigations are necessary it is upto the radiologist to suggest and carry out. If he claims to be a consultant, as he has a right to do, he must prove worthy of the trust and justify the position and prestige he enjoys.

Regarding the vexed question of the ownership of radiograms, there is a false notion amongst the public that because they have paid for the investigations, the films are their property. It must be emphasised that this is not so and that the radiograms are the property of the hospitals or of the radiologist who carries out the examination. This has been established in the Court of Law time and again. Several law suits are recorded in medical journal where this point has been unequivocally decided. Unfortunately the practice in this country is to hand over the films to the patients, a very pernicious system which has existed from the very beginning. The correct procedure should be for the radiologist to send the report to the doctor referring the case, to whom the films may be send if asked for. The referring doctor's desire to see the films is quite understandable. Apart from the scientist interest, the physicians and the surgeons may want to see with the view to gauge the specific characteristic of the diseased processes, if it is a case of fractures, the type of fracture, whether there is deformity or otherwise. On the basis of such visualisation as depicted in the radiograms, depends the treatment of the patients.

A very objectionable feature in radiological feature in the radiological practice at the present moment is the system of the selling films introduced by private hospitals. They take up investigation of outside cases and charge them a specified fee per films or for screening, making it a commercial transaction. It is true that in taking up cases referred by outside practitioners they are doing a distinct service to the public, as a matter of fact there can be no better place than a hospital for such investigations with it's superior equipment and specialised staff. The objectionable part is the commercialisation involved in wantonly giving away the films with the express purpose of deriving a substantial income. We sincerely hope the hospitals will stop such unethical practices, derogatory to the dignity of our profession.

